# An observational study of the lung clearance index throughout childhood in cystic fibrosis: early years matter

**DOI:** 10.1183/13993003.00006-2020

**Published:** 2020-10-01

**Authors:** Gwyneth Davies, Sanja Stanojevic, Emma Raywood, Julie A. Duncan, Janet Stocks, Sooky Lum, Andrew Bush, Laura Viviani, Angie Wade, Alistair Calder, Catherine M. Owens, Christophe Goubau, Siobhán B. Carr, Cara J. Bossley, Caroline Pao, Paul Aurora

**Affiliations:** 1 Infection, Immunity and Inflammation Research and Teaching Dept, UCL Great Ormond Street Institute of Child Health (UCL GOS ICH), London, UK; 2 Dept of Respiratory Medicine, Great Ormond Street Hospital for Children NHS Foundation Trust, London, UK; 3 Translational Medicine, SickKids Research Institute, Toronto, ON, Canada; 4 Dept of Paediatric Respiratory Medicine, Imperial College and Royal Brompton and Harefield Hospital NHS Foundation Trust, London, UK; 5 Clinical Epidemiology, Nutrition and Biostatistics Section, UCL GOS ICH, London, UK; 6 Dept of Radiology, Great Ormond Street Hospital for Children NHS Foundation Trust, London, UK; 7 Dept of Paediatric Respiratory Medicine, Kings College Hospital, London, UK; 8 Dept of Paediatric Respiratory Medicine, Royal London Hospital, London, UK; 9 Joint first authors

## Abstract

The London Cystic Fibrosis Collaboration (LCFC) has prospectively followed a clinically diagnosed cohort of infants with cystic fibrosis (CF) born in South East England since 1999 [1–4]. Over the past 20 years, the LCFC has obtained comprehensive measures of lung function and structure, including measures of ventilation inhomogeneity (lung clearance index (LCI)) and high-resolution computed tomography (HRCT) scans. By pre-school age, 73% of this cohort had LCI above the limits of normal, compared with 7% with abnormal forced expiratory volume in 0.5 seconds (FEV_0.5_) [1]. Children with elevated LCI during pre-school years also had worse lung function at early school age [2]. The aim of this study was to investigate how LCI changes across childhood to better understand to what extent LCI results at pre-school age are an indicator of lung disease severity in adolescence.

## Abstract 2

**Lung clearance index (LCI) in the early years was associated with LCI during adolescence in children with cystic fibrosis. Pre-school LCI may help to identify children in whom treatment could be intensified.** https://bit.ly/2yKyMbM

*To the Editor*:

The London Cystic Fibrosis Collaboration (LCFC) has prospectively followed a clinically diagnosed cohort of infants with cystic fibrosis (CF) born in South East England since 1999 [1–4]. Over the past 20 years, the LCFC has obtained comprehensive measures of lung function and structure, including measures of ventilation inhomogeneity (lung clearance index (LCI)) and high-resolution computed tomography (HRCT) scans. By pre-school age, 73% of this cohort had LCI above the limits of normal, compared with 7% with abnormal forced expiratory volume in 0.5 seconds (FEV_0.5_) [1]. Children with elevated LCI during pre-school years also had worse lung function at early school age [2]. The aim of this study was to investigate how LCI changes across childhood to better understand to what extent LCI results at pre-school age are an indicator of lung disease severity in adolescence.

Details of the LCFC cohort have been previously published [1, 4, 5]. Starting in 2013, adolescents with CF and their contemporaneous controls, aged 12–17 years, were recruited for follow-up. Anthropometric measurements, clinical examination and lung function testing were performed on a single day, along with HRCT in adolescents with CF. Inclusion criteria included clinical stability at the time of test (no change in respiratory symptoms or medication within the previous 2 weeks). Ethical approval was granted from the NHS Health Research Authority National Research Ethics Service (reference 13/LO/0322) and written informed consent obtained.

Multiple breath washout (MBW) was performed using a mass spectrometer with SF_6_ as the tracer gas [2, 3]. Apart from a face mask being used for pre-school and mouthpiece for older subjects, identical equipment was used as for previous study visits. Quality control was undertaken to ensure the same settings and algorithms were applied for all study visits. An elevated LCI was defined as ≥1.96 z-scores using published reference equations [6]. Spirometry was performed after MBW [7]. Forced expiratory volume in 1 s (FEV_1_) was the primary outcome. Abnormal FEV_1_ was defined as ≤−1.96 z-scores (lower limit of normal (LLN)) using the 2012 Global Lung Function Initiative (GLI) reference equations [8]. For longitudinal analysis of FEV, z-scores for FEV at time ‘t’ in pre-school children were calculated from FEV_0.75_ if FEV_1_ was not available (zFEV_t_). Anthropometric z-scores were calculated from the British 1990 growth charts [9].

Low-dose (100 kV) volumetric CT scans were acquired at total lung capacity, followed by three additional expiratory scans, in non-contiguous fashion performed at pre-determined levels. CT scans were scored using Brody-II [10], blinded to any previous results or current clinical condition, by the same radiologist who had scored the school-age scans. The total Brody-II CF-CT score was used as the primary outcome, with secondary outcomes being the sub-scores of bronchiectasis, peribronchial thickening and air trapping.

Mixed-effects linear regression with random intercepts and slopes was used to determine the rate of LCI change through childhood using all available data from the LCFC. An exchangeable correlation structure was used, and an interaction between age and group was used to determine whether the rate of change differed between the healthy and CF groups. Linear regression was used to estimate the factors (determined *a priori*) associated with FEV_1_ z-scores during adolescence. The same analysis was repeated using LCI and CT at adolescence as outcomes. Statistical analysis was performed using STATA (Version 15.0).

43 subjects with CF tested at pre-school were followed up to adolescence; four subjects had either died or undergone lung transplantation. In those who died (one post-lung transplant, one following assessment for urgent lung transplantation and one unknown), the reported LCI values at pre-school were 11.4, 8.0 and 7.3, with corresponding zFEV_t_: −3.5, 0.1 and −1.0. In the surviving lung transplant subject, pre-school LCI was 11.92 and zFEV_1_ −1.1.

Anthropometrically, the CF group included in these analyses was similar to contemporaneous controls during pre-school years, whereas by adolescence, the controls were significantly taller and heavier, although there was no significant difference in zBMI. 37% (13/35, 8 unknown) CF subjects were positive for *Pseudomonas aeruginosa* within 12 months prior to their pre-school test, and similarly, 41% at adolescent follow-up (17/41, 2 unknown). Subjects with CF had significantly lower zFEV_1_ compared with the healthy group at adolescent follow-up (mean difference −1.25 (95% CI −1.86; −0.65), but only 30% had a zFEV_1_ value below the LLN. LCI was more than 3 units higher in the CF group compared with the healthy adolescents (mean difference 3.22, 95% CI 2.29; 4.16). The median total Brody-II CF-CT score was 19 (interquartile range 10–46); range 0–102 (maximum possible score of 243).

LCI increased (deteriorated) with age in the CF group by 0.18 units per year (95% CI 0.14; 0.21; intra-class correlation 0.67). While there was a small, statistically significant, increase in LCI in the control group with age, this was significantly faster in CF (figure 1). The majority (88%) of the CF subjects had abnormal LCI at adolescence; using published upper limits of normal [6], pre-school LCI had a sensitivity of 77% (95% CI 58.9; 90.4) and a specificity of 66% (95% CI 9.43; 99.2), positive predictive value 96% (95% CI 79.6; 99.9) and negative predictive value 22.2% (95% CI 2.81; 60.0) (area under the curve 0.72 (95% CI 0.39; 1.0)) to predict abnormal LCI at adolescence. FEV_t_ at pre-school had a similar sensitivity (80% (95% CI 44.4; 97.5) but a much lower specificity for abnormal LCI at adolescence (27%; 95% CI 11.6; 47.8).

**FIGURE 1:**
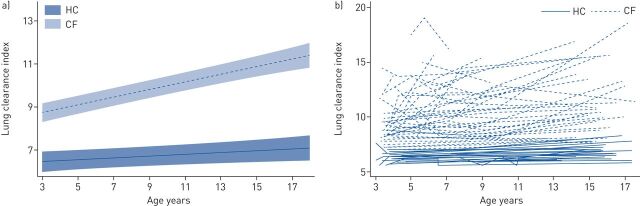
Change in lung clearance index (LCI) with age in healthy subjects (HC) and those with cystic fibrosis (CF) throughout childhood. a) Rate of change in LCI with age in health (slope 0.04 (95% CI 0.002; 0.08) and CF (slope 0.18 (95% CI 0.14; 0.21). The rate of change in CF was significantly different from the healthy group (interaction coefficient −0.05 (95% CI −0.09; −0.002). Results represent mean LCI with 95% confidence intervals for the CF group (dashed line, pale shaded area) and contemporaneous controls (solid line, darker shaded area), respectively. b) Individual changes in LCI in health (solid line) and CF (dashed line).

There were no statistically significant associations between demographic characteristics (sex, genotype, age at diagnosis, age at pre-school visit) and adolescent outcomes (zFEV_1_, LCI and CT score), nor was there a statistically significant association with the acquisition of *P. aeruginosa* prior to their pre-school visit. Pre-school LCI was positively associated with adolescent LCI (slope 0.97 (95% CI 0.50; 1.44), n=34) and adolescent CT (6.22 (1.5; 10.9), n=33), but not adolescent zFEV_1_ (−0.27 (−0.54; 0.001), n=43). In comparison, pre-school FEV_1_ was associated with adolescent zFEV_1_ (0.39 (0.02; 0.76), n=38) but not adolescent LCI (−0.73 (−1.54; 0.08), n=35) or CT (−0.89 (−8.7; 6.9), n=34). Similar results were observed for the association between pre-school LCI and adolescent bronchiectasis, bronchial wall thickness and air trapping sub-scores of the Brody-II CF-CT.

Our findings confirm that LCI at pre-school age correlates with LCI at adolescence in clinically diagnosed children with CF. Higher LCI values during pre-school are associated with worse lung function and structure during adolescence. Together with the finding that LCI is more sensitive than spirometry during pre-school years, these longitudinal data confirm that LCI is a useful tool to identify young children who may benefit from intensified clinical intervention.

In this observational cohort, an elevated LCI during pre-school years was an indicator of worse lung disease throughout childhood and adolescence. This is highly relevant to current populations of children with CF, even though most will now have been diagnosed by newborn screening (NBS). Although more recent, newborn screened, cohorts have milder phenotypes, there remain individuals with elevated LCI at pre-school age [11], and our data suggest this is of prognostic significance. More recent pre-school studies in NBS subjects have also identified this period as a critical window for monitoring early lung disease in children with CF; the LCI can detect significant lung function deterioration, and was associated with acute worsening during pulmonary exacerbations [12]. In addition, the SHIP (Saline Hypertonic in Preschoolers) study suggests that LCI is responsive to treatment in this age group [13]. Thus, using LCI to identify the deterioration of lung function in early childhood may lead to early intervention to alter the course of lung disease, and long-term outcomes.

This is the first study to report that pre-school LCI is associated with chest CT structural abnormalities in adolescence. This is consistent with a cross-sectional study which reported significant associations between school-age (range 7–16 years; mean 9.8 years) LCI and extent of total disease, bronchiectasis and air trapping on chest CT in an Australian NBS cohort [14].

A major strength of this study is the prospective follow-up when patients were clinically stable, with measurements performed at a single centre, and the inclusion of a contemporaneous healthy control group measured using the same equipment and protocol. Despite the small number of subjects overall, this is a unique cohort with comprehensive physiology outcomes. In this study, the same radiologist used the Brody-II CF-CT to score scans at school age and adolescence, to facilitate comparison of results. While newer CF scoring systems are now available [15], we felt it inappropriate to change pre-specified analysis plans. The long interval between study visits meant that only limited clinical data were captured at the study visits, with potential for missing data. Furthermore, since clinicians were not blinded to lung function results, they may have changed management. The unexpected rise in LCI in healthy adolescents could either indicate natural increases in LCI in health [16] or a potential bias which may have increased the number of adolescents identified with abnormal LCI, but neither would have influenced conclusions regarding the relative changes in LCI over time.

LCI measured during the pre-school years correlates with LCI at adolescence and is associated with chest CT abnormalities at adolescence. Elevated pre-school LCI is an ominous warning of impaired adolescent lung function in clinically diagnosed children with CF, and further emphasises a window of opportunity to intervene at an early age to improve outcomes.

## Shareable PDF

10.1183/13993003.00006-2020.Shareable1 This one-page PDF can be shared freely online.
Shareable PDF ERJ-00006-2020.Shareable
