# Supplementary material for: An observational study of the lung clearance index throughout childhood in cystic fibrosis: early years matter
Source: Eur Respir J. 2020 Oct 1;56(4):2000006. doi: 10.1183/13993003.00006-2020 (PMC7527650; doi:10.1183/13993003.00006-2020)
Supplement: Supplementary file 1 [file ERJ-00006-2020.Shareable.pdf]

SHAREABLE PDF

An observational study of the lung clearance index throughout childhood in cystic fibrosis: early years matter

Gwyneth Davies [1,2,9], Sanja Stanojevic[3,9], Emma Raywood [1], Julie A. Duncan[1], Janet Stocks[1], Sooky Lum[1], Andrew Bush[4], Laura Viviani[1], Angie Wade[5], Alistair Calder[6], Catherine M. Owens[6], Christophe Goubau[1], Siobhán B. Carr [4], Cara J. Bossley[7], Caroline Pao[8] and Paul Aurora[1,2] on behalf of the London Cystic Fibrosis Collaboration

Affiliations: [1]Infection, Immunity and Inflammation Research and Teaching Dept, UCL Great Ormond Street Institute of Child Health (UCL GOS ICH), London, UK. [2]Dept of Respiratory Medicine, Great Ormond Street Hospital for Children NHS Foundation Trust, London, UK. [3]Translational Medicine, SickKids Research Institute, Toronto, ON, Canada. [4]Dept of Paediatric Respiratory Medicine, Imperial College and Royal Brompton and Harefield Hospital NHS Foundation Trust, London, UK. [5]Clinical Epidemiology, Nutrition and Biostatistics Section, UCL GOS ICH, London, UK. [6]Dept of Radiology, Great Ormond Street Hospital for Children NHS Foundation Trust, London, UK. [7]Dept of Paediatric Respiratory Medicine, Kings College Hospital, London, UK. [8]Dept of Paediatric Respiratory Medicine, Royal London Hospital, London, UK. [9]Joint first authors.

Correspondence: Gwyneth Davies, Infection, Immunity and Inflammation Research and Teaching Dept, UCL Great Ormond Street Institute of Child Health, 30 Guilford Street, London WC1N 1EH, UK. E-mail: gwyneth.davies@ucl.ac.uk.

@ERSpublications
Lung clearance index (LCI) in the early years was associated with LCI during adolescence in children with cystic fibrosis. Pre-school LCI may help to identify children in whom treatment could be intensified. https://bit.ly/2yKyMbM

Cite this article as: Davies G, Stanojevic S, Raywood E, *et al.* An observational study of the lung clearance index throughout childhood in cystic fibrosis: early years matter. *Eur Respir J* 2020; 56: 2000006 [https://doi.org/10.1183/13993003.00006-2020].

This single-page version can be shared freely online.

To the Editor:

The London Cystic Fibrosis Collaboration (LCFC) has prospectively followed a clinically diagnosed cohort of infants with cystic fibrosis (CF) born in South East England since 1999 [1–4]. Over the past 20 years, the LCFC has obtained comprehensive measures of lung function and structure, including measures of ventilation inhomogeneity (lung clearance index (LCI)) and high-resolution computed tomography (HRCT) scans. By pre-school age, 73% of this cohort had LCI above the limits of normal, compared with 7% with abnormal forced expiratory volume in 0.5 seconds ($FEV_{0.5}$) [1]. Children with elevated LCI during pre-school years also had worse lung function at early school age [2]. The aim of this study was to investigate how LCI changes across childhood to better understand to what extent LCI results at pre-school age are an indicator of lung disease severity in adolescence.

Copyright ©ERS 2020.. This version is distributed under the terms of the Creative Commons Attribution Licence 4.0.
